# Supplementary material for: Z3-vestigial nematic order due to superconducting fluctuations in the doped topological insulators NbxBi2Se3 and CuxBi2Se3
Source: Nat Commun. 2020 Jun 16;11:3056. doi: 10.1038/s41467-020-16871-9 (PMC7298044; doi:10.1038/s41467-020-16871-9)
Supplement: Supplementary file 1 — Supplementary Information [file 41467_2020_16871_MOESM1_ESM.pdf]

Supplementary Information

Z_3-vestigial nematic order due to superconducting fluctuations in the doped topological insulators $Nb_xBi_2Se_3$ and $Cu_xBi_2Se_3$

by Cho et al.

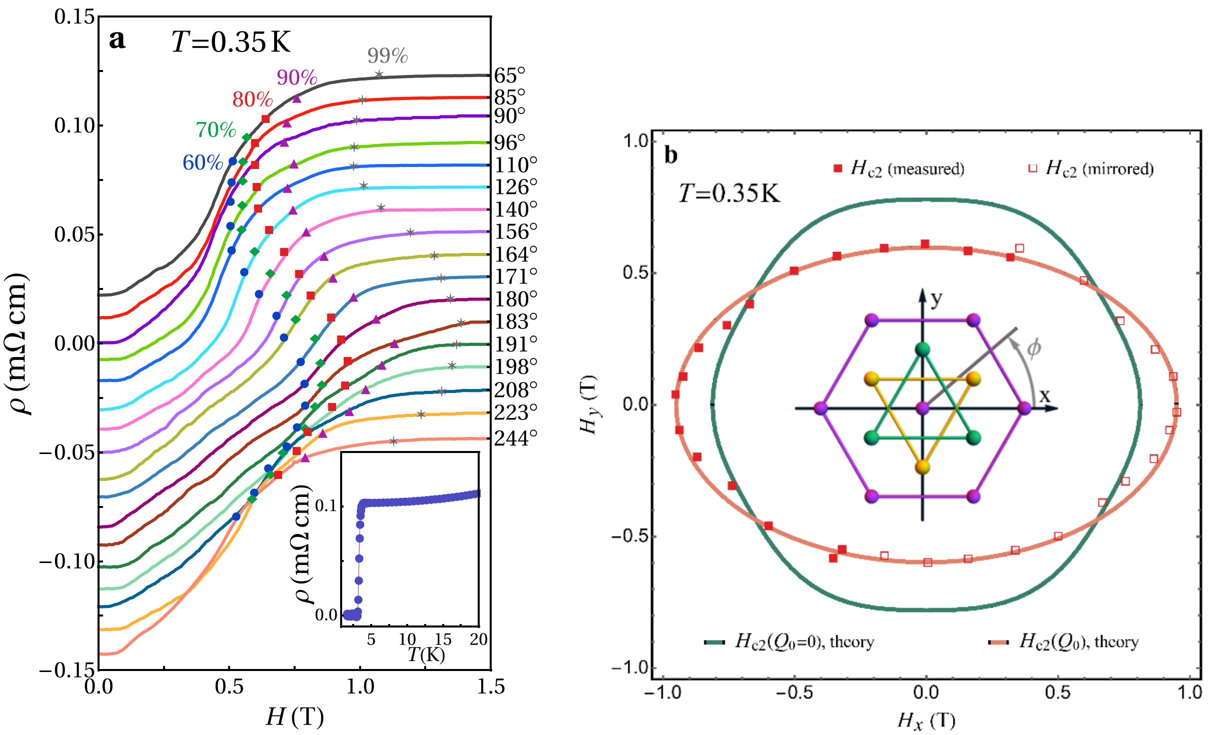

Supplementary Figure 1 Dependence of the upper critical field H_{c2} of an additional $Nb_{0.25}Bi_2Se_3$ single crystal (Sample 2) on the angle ϕ. (a) Magnetoresistance data taken at 350 mK for various alignments of the magnetic field in the Bi_2Se_3 basal plane. The additional symbols mark the characteristic fields in which the magnetoresistance reaches 60%, 70%, 80%, 90% and 99% of the normal state value. The data were shifted vertically by -0.01 mΩ cm for better clarity, except for the $\phi = 90°$ data. **(b)** Polar plot of characteristic fields where the magnetoresistance reaches 70% of the normal state value, depending on the field direction in the Bi_2Se_3 basal plane. Only the full squares are real data, while the open squares are the same data shifted by 180° to better illustrate the full angular dependence. In the center, the corresponding crystal structure is added. The lines are theoretical expectations of a superconductor with trigonal symmetry without (green) and with (orange) vestigial nematic order**.**

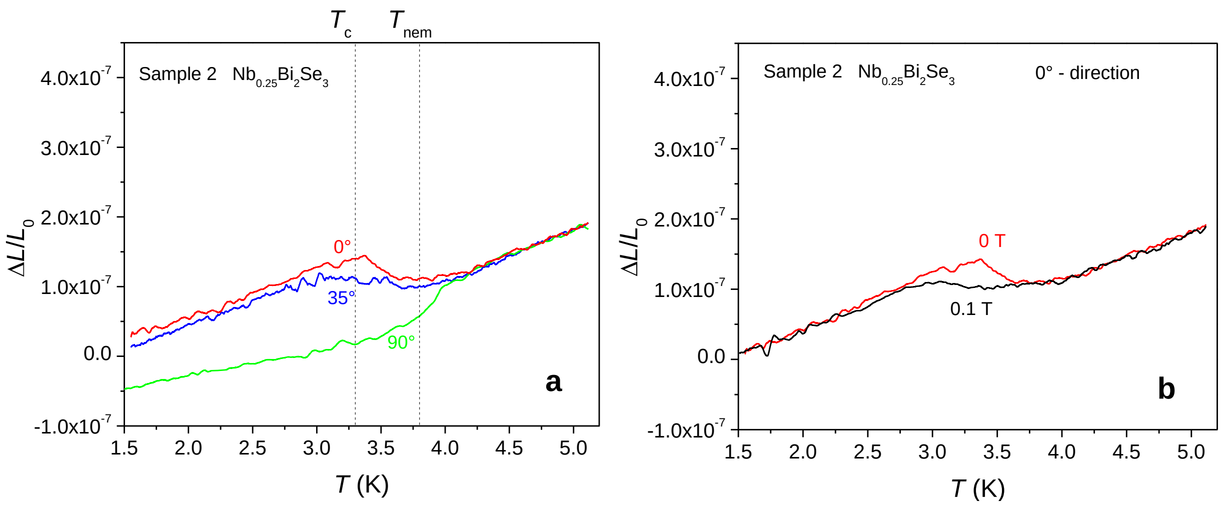

Supplementary Figure 2 Thermal expansion of $Nb_{0.25}Bi_2Se_3$ (Sample 2). (a) The linear thermal expansion $\Delta L(T)/L_0$ measured in three directions in the Bi_2Se_3 basal plane corresponding to 0°, 35° and 90°, A pronounced anisotropy appears in $\Delta L(T)/L_0$ below $T_{\mathrm{nem}} \approx 3.8$ K. **(b)** $\Delta L(T)/L_0$ measured along the 0° direction in zero field (same data as in **a**) and in an applied magnetic field of 0.1 T.

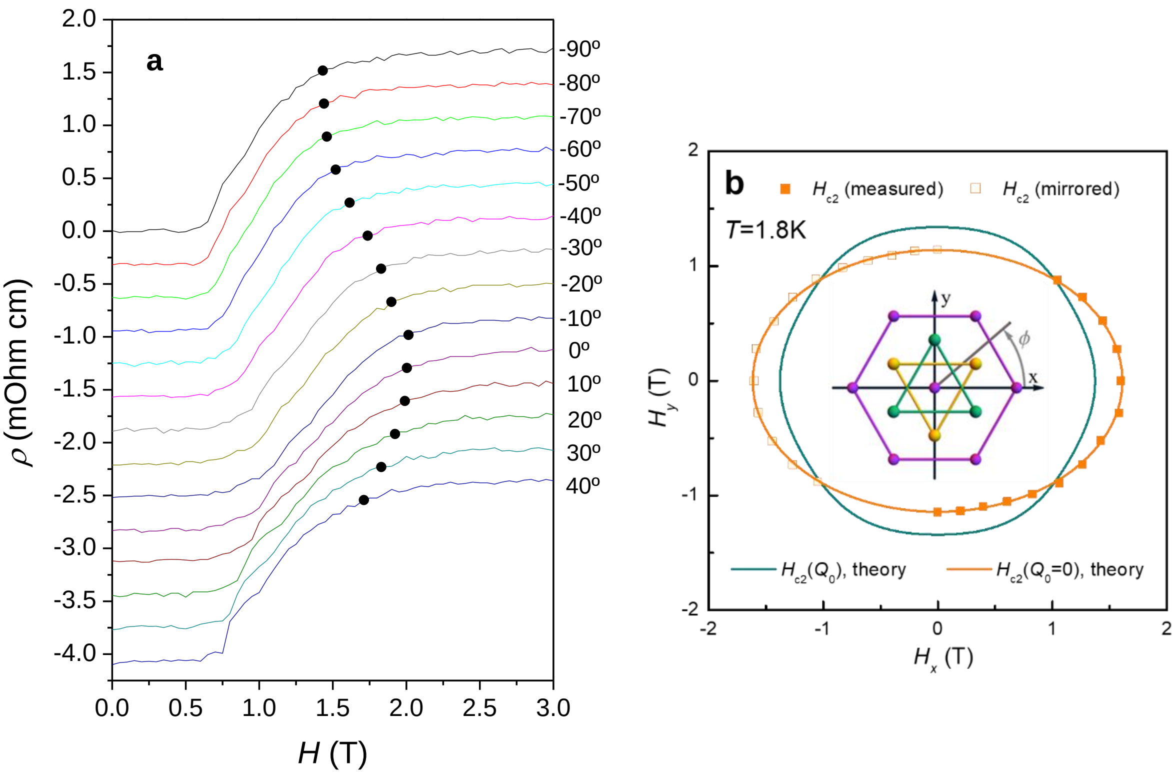

Supplementary Figure 3 Dependence of the upper critical field H_{c2} of $Cu_{0.2}Bi_2Se_3$ on the angle ϕ. (a) Field-angle-resolved magnetoresistance data taken at 1.8 K (Sample 3) for various alignments of the magnetic field in the Bi_2Se_3 basal plane. The additional circles mark the characteristic fields in which the magnetoresistance reaches 90% of the normal state value. The data were shifted vertically by -0.3 mΩ cm for better clarity, except for the $\phi = -90°$ data. **(b)** Polar plot of characteristic fields where the magnetoresistance reaches 90% of the normal state value, depending on the field direction in the Bi_2Se_3 basal plane. Only the full squares are real data, while the open squares are the same data shifted by 180° to better illustrate the full angular dependence. In the center, the corresponding crystal structure is added. The lines are theoretical expectations of a superconductor with trigonal symmetry without (green) and with (orange) vestigial nematic order.

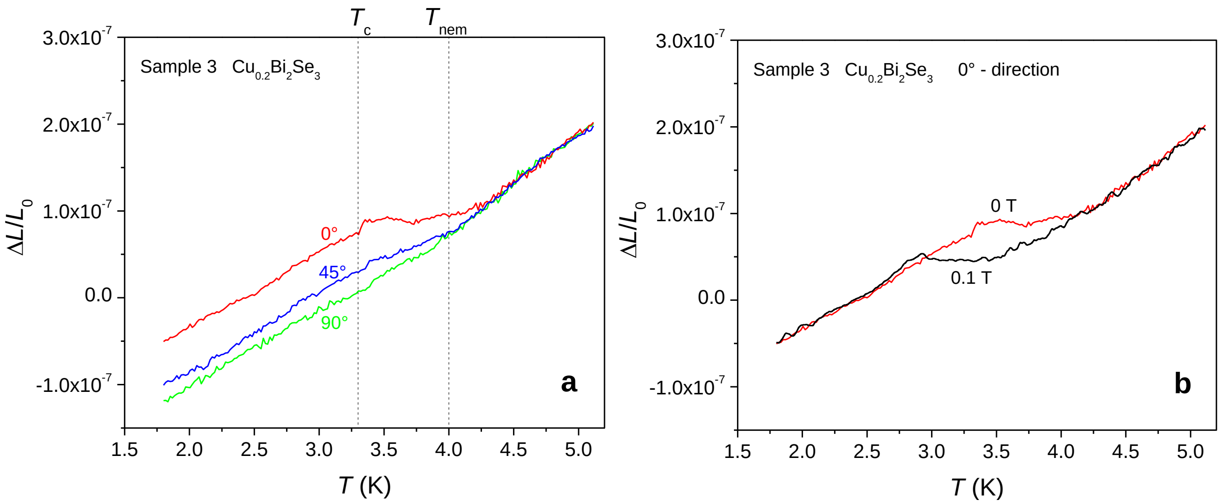

Supplementary Figure 4 Thermal expansion of $Cu_{0.2}Bi_2Se_3$ (Sample 3). (a) Linear thermal expansion $\Delta L(T)/L_0$ measured in three directions in the Bi_2Se_3 basal plane corresponding to 0°, 45° and 90°, A pronounced anisotropy appears in $\Delta L(T)/L_0$ below $T_{\mathrm{nem}} \approx 4.0$ K. T_c is marked according to the arrows in Supplementary Figure 5 c & d. **(b)** $\Delta L(T)/L_0$ measured along the 0° direction in zero field (same data as in **a**) and in an applied magnetic field of 0.1 T.

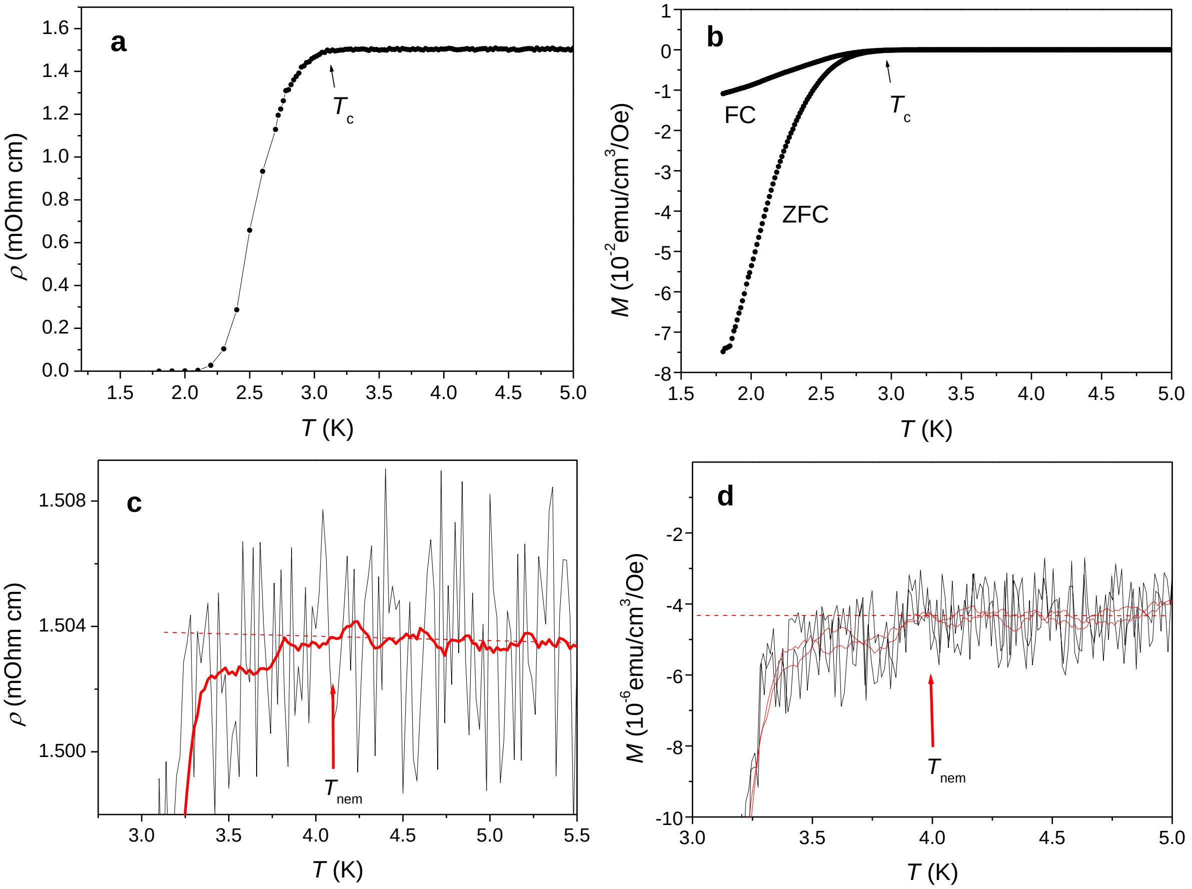

Supplementary Figure 5 Electric resistivity and DC magnetization of $Cu_{0.2}Bi_2Se_3$ (Sample 3). (a) The resistivity shows the main superconducting transition at T_c. **(b)** DC magnetization measured under zero field cooled (ZFC) and field cooled (FC) conditions, which shows the main superconducting transition at T_c. **(c)** Magnification of the onset of the superconducting transition in resistivity, illustrating that a weak downturn below T_{nem} = 4.0 K occurs. The black scattered data are the original data and the thick red line is data smoothed over 25 points for better clarity. **(d)** Magnification of the onset of the superconducting transition in the DC magnetization, showing that a weak Meissner signal already occurs below T_{nem} = 4.0 K. The black scattered data are the original data and the thick red line is data smoothed over 25 points.

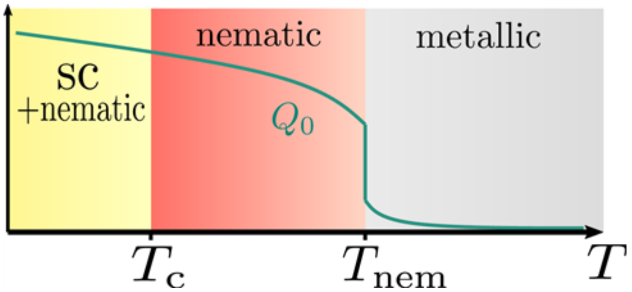

Supplementary Figure 6 Nematic order parameter of a sample with net internal strain. Doped Bi_2Se_3 displays for a given sample always the same nematic axis, even though spontaneous symmetry breaking should yield all three degenerate vales of the nematic orientation. A natural explanation are random internal strain fields that do not average to zero. Then the overall direction is set above T_{nem}, while there can still be a pronounced transition at this temperature. Strain fields act like a tiny magnetic field in a ferromagnet that will always tip the balance between otherwise degenerate magnetization directions.

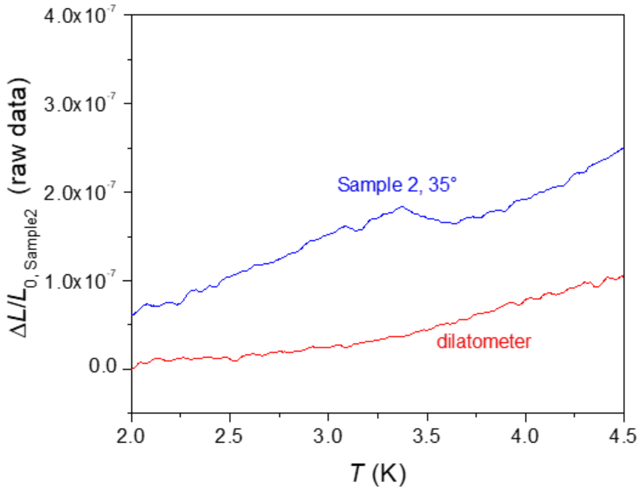

Supplementary Figure 7 Comparison of $\Delta L/L_0$ raw data with an empty measurement. The blue data was taken with Sample 2, whereas for the empty measurement (red data) the screw that normally presses the sample against one of the capacitor plates was pressed directly against the plate without sample. This demonstrates that the observed anomaly at T_{nem} is not due to the background signal of the dilatometer.

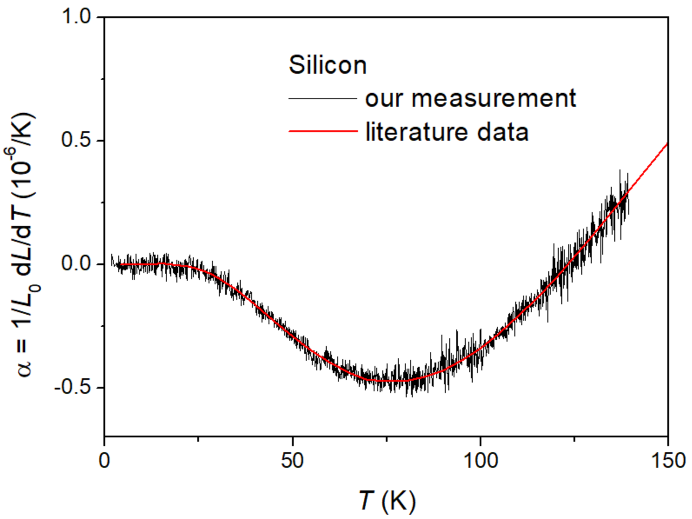

Supplementary Figure 8 Calibration measurement on a Si single crystal. Here we show the linear thermal expansion coefficient $\alpha(T) = 1/L_0\ \mathrm{d}L(T)/\mathrm{d}T$. The measured data agree well with literature data[1]. The length L_0 of the undoped Si crystal was 2 mm.

Supplementary Note 1

Additional data from another $Nb_{0.25}Bi_2Se_3$ single crystal (Sample 2)

Sample 2 is a $Nb_{0.25}Bi_2Se_3$ single crystal from the same batch as Sample 1, for which the data is included in the main article. The superconducting transition in zero field is slightly broader (see inset of Supplementary Figure 1a) and in a magnetic field the upper critical field transition is quite wide, but with a certain substructure (Supplementary Figure 1a). In the specific heat there is a sequence of three transitions, which indicates that there are two minority domains in addition to a majority domain, in which the nematic order parameter is rotated relative to the majority domain. Nevertheless, the presence of a majority domain allows a qualitative comparison of its nematic characteristics with Sample 1, although the overall nematic effects will be weaker due to these twinning effects. Supplementary Figure 1a shows the magnetoresistance of Sample 2, measured at T= 0.35 K for different magnetic field directions in the Bi_2Se_3 basal plane. ‘Regardless of the broadening, a clear two-fold symmetry of the H_{c2} transition can be seen, which is further illustrated in the polar plot in Supplementary Figure 1b. The anisotropy is weaker than in Sample 1.

Supplementary Figure 2a shows the thermal expansion $\Delta L/L_0$ in zero magnetic field, measured in three directions in the Bi_2Se_3 basal plane: ϕ = 0°, 35° and 90°. A distinct splitting of the data is visible slightly below 4 K, with the sample expanding along the 0° and 35° directions but shrinking along the 90° direction. The overall behavior is similar to Sample 1, although the splitting of the data is weaker, probably due to contributions of the two minority domains with different orientation of the nematic order parameters, which partially cancel each other out with the contribution of the majority domain. Moreover, the smaller anomalies at T_c are too small to be resolved here.

Supplementary Figure 2b shows the same zero-field data for the 0° direction together with data for the same direction measured in an applied field of 0.1 T. It is obvious that the transition anomaly is shifted to lower temperatures. This shows a close connection of the nematic transition with the superconducting order and further supports that it is triggered by the onset of the superconducting fluctuations.

Supplementary Note 2

Additional data from a $Cu_{0.2}Bi_2Se_3$ single crystal (Sample 3)

Sample 3 is a $Cu_{0.2}Bi_2Se_3$ single crystal. Supplementary Figure 3a shows the magnetoresistance measured at T= 1.8 K for various magnetic fields applied along different directions in the Bi_2Se_3 plane. For this sample, too, a clear two-fold symmetry of the H_{c2} transition can be seen, which is further illustrated in the polar plot in Supplementary Figure 3b. Again, the anisotropy is weaker than in Sample 1, but this may be at least partly a consequence of the higher temperature at which these measurements were made.

Supplementary Figure 4a shows the thermal expansion $\Delta L/L_0$ in zero magnetic field, measured along three directions in the Bi_2Se_3 basal plane: ϕ = 0°, 45° and 90°. A deviation of the three datasets is visible below ~4 K, with the sample expanding in the 0° and 45° directions, but shrinking in the 90° direction. The overall behavior is similar to Sample 1 and 2, although the deviation of the datasets is weaker, possibly due to a lower superconducting volume fraction or charge density. The smaller anomalies at T_c are too small to be resolved clearly, although there are some tiny anomalies around 3.3 K that may indicate the main superconducting transition.
Supplementary Figure 4b shows the same data for the 0° direction together with data for the same direction measured in an applied field of 0.1 T. It is obvious that the transition anomaly is shifted to lower temperatures, similar to what we observed for Sample 2.

Supplementary Figure 5 shows zero-field electric resistivity data (**a**,**c**) and DC magnetization data (**b**,**d**) for Sample 3. Panel 5a and 5b show the entire data range, while c and d show an enlargement of the onset of the superconducting transition. According to panel 5a and 5b, the superconducting transition appears somewhere around 3 K, but it is difficult to define a sharp transition temperature because the transition is quire wide and continuous. In the enlarged plots in Supplementary Figure 5c & 5d, at 3.3 K there is a sharp bend with pronounced downturn below. This is also the temperature at which the tiny anomalies occur in the thermal expansion shown in Supplementary Figure 4a, which is probably best defined as T_c. The enlarged data in 5c and 5d show that a certain reduced resistivity and a weak Meissner effect up to 4.0 K remain, similar to what was observed in Sample 1. The splitting of the thermal expansion data along different directions develops in the same temperature range. This further supports our interpretation of a vestigial nematic order. The similarity of our $Cu_{0.2}Bi_2Se_3$ data with our $Nb_{0.25}Bi_2Se_3$ data suggests that vestigial nematic order is universally found in superconducting doped Bi_2Se_3.

Supplementary Discussion

Nematic transition and internal strain fields

One of the puzzles of nematicity in doped Bi_2Se_3 has been the fact that the two-fold rotation axis of a given crystal remains always the same, even after a thermal cycle well above the transition temperature. If there was true spontaneous symmetry breaking, one would expect that the three degenerate nematic axes occur with equal probability. On the one hand, nematicity becomes really only visible at, or – as we demonstrate – near T_c, i.e. it is tied to superconductivity in some way. On the other hand, the axis of spontaneous symmetry breaking seems to be set much above T_c. However, this behavior does not rule out a superconducting origin for nematicity. In a way the system behaves similar to a tiny magnetic field that always induces the same magnetization direction of a ferromagnet. The magnetization only becomes sufficiently large to be observable near the zero field Curie temperature. In our case the role of the magnetic field is played by strain fields.

A natural explanation for the observed behavior is that random internal strain fields do not average to zero and tip the balance between the otherwise degenerate nematic states. These strain fields seem therefore extrinsic and not related to the low-temperature physics that governs the superconducting state. Notice, for a trigonal crystal any non-symmetric strain, even along the c-axis, induces a finite nematic order parameter. In Supplementary Figure 6 we show that this is not necessarily in contradiction with a sharp nematic phase transition. This is due to the fact that the transition is of first order. While the first order transition of the three-state Potts model with short range interactions would be rounded by random-field disorder with finite disorder correlation length, it is not clear whether the same is true if one includes long-ranged strain interactions.

To summarize, the origin of internal strain field remains puzzling, but such random strain explains the fact that the direction of the spontaneous symmetry breaking is fixed for a given crystal. The origin and magnitude for the intrinsic nematicity seem to be weakly affected by these strain fields.

Supplementary References

1. Lyon, K. G., Salinger, G. L., Swenson, C. A. Linear thermal expansion measurements on silicon from 6 to 340 K, J. App. Phys. **48,** 865 (1977).
